# Genomic Insights into *Stutzerimonas kunmingensis* TFRC-KFRI-1 Isolated from Manila Clam (*Ruditapes philippinarum*): Functional and Phylogenetic Analysis

**DOI:** 10.3390/microorganisms12122402

**Published:** 2024-11-23

**Authors:** Myunglip Lee, Sunghun Yi, Jiho Choi, Yukyoung Pak, Chaehyeon Lim, Yucheol Kim

**Affiliations:** 1 Korea Food Research Institute (KFRI), Nongsaengmyeong-ro, Iseo-myeon, Wanju-gun 55365, Jeollabuk-do, Republic of Korea; l.myunglip@kfri.re.kr (M.L.);; 2 National Institute of Fisheries Science (NIFS), 405, Gangbyeon-ro, Gunsan-si 54042, Jeonbuk-do, Republic of Korea; 3 Genetics and Breeding Research Center, National Institute of Fisheries Science, Geoje 53334, Republic of Korea

**Keywords:** *Stutzerimonas kunmingensis*, genome sequencing, probiotics, carbohydrate degradation, Manila clam

## Abstract

*Stutzerimonas kunmingensis* TFRC-KFRI-1, isolated from the gut of Manila Clam in the sediment of the West Sea of Korea, was investigated for its potential as a probiotic bacterium. This strain, belonging to the family *Pseudomonadaceae*, was previously classified as *Pseudomonas kunmingensis* but later reclassified to the genus *Stutzerimonas*, known for species with bioremediation and probiotic properties. To evaluate its genomic features and potential applications, we performed draft-genome sequencing and analysis. The genome of *S. kunmingensis* TFRC-KFRI-1 was assembled into a 4,756,396 bp sequence with a 62.8% GC content. Genomic analysis suggested potential genes for carbohydrate degradation and lactic acid production. The strain exhibited high average nucleotide identity (ANI) and 16S rRNA similarity with *S. kunmingensis* HL22-2^T^, further supporting its potential as a probiotic. This genome sequence provides valuable insights into the functional capabilities of *S. kunmingensis* TFRC-KFRI-1 and its potential applications in various industries, including aquaculture and food biotechnology. The genome sequence is available under GenBank accession number JBGJJB000000000.1, with related project information under BioProject PRJNA1147901 and Bio-Sample SAMN43173893.

## 1. Introduction

Probiotics are live microorganisms that, when administered in adequate amounts, confer health benefits to the host [1]. They have gained significant attention in healthcare, veterinary medicine, and aquaculture due to their diverse applications and health-promoting properties [2]. With the rise of antibiotic resistance and an increasing demand for sustainable agricultural and food production practices, probiotics are increasingly viewed as an alternative solution [3,4].

In aquaculture, probiotics offer a natural, non-chemical method to enhance growth performance, feed efficiency, and immune responses in aquatic organisms, providing a sustainable alternative to antibiotics [5]. The application of probiotics in aquaculture can alleviate the environmental and health risks associated with the overuse of antibiotics, promoting a more sustainable and responsible approach to aquatic animal production.

The genus *Stutzerimonas*, belonging to the family *Pseudomonadaceae*, includes strains that were previously classified within the *Pseudomonas stutzeri* phylogenetic group based on genetic analysis, and consists of Gram-negative bacteria commonly found in soil, water, and marine sediments [6,7].

Certain *Stutzerimonas* species are well studied for their bioremediation potential due to their capacity to degrade harmful environmental pollutants, including aromatic compounds and heavy metals [8,9]. This adaptability indicates that *Stutzerimonas* species may survive and thrive in the gastrointestinal tracts of aquatic animals, making them promising probiotic candidates.

Recent research has focused on the probiotic potential of *Stutzerimonas*. Like other established probiotic genera, members of this genus have shown promise in modulating immune function and contributing to gastrointestinal health [10,11]. For instance, some *Stutzerimonas* strains exhibit remarkable resilience in extreme environments, such as deep-sea habitats, suggesting robust survival capabilities and the potential to withstand harsh conditions in the gastrointestinal tract [12,13]. However, the probiotic potential of *Stutzerimonas* remains largely unexplored, necessitating further research to assess its applicability in various fields, including aquaculture.

This study examines *Stutzerimonas kunmingensis* TFRC-KFRI-1, a strain isolated from the gut of the Manila clam (*Ruditapes philippinarum*) in the West Sea of Korea. We conducted genome sequencing and analysis to elucidate its genomic features and assess its probiotic potential, focusing on potential applications in aquaculture. Our aim was to characterize the genomic features of *S. kunmingensis* TFRC-KFRI-1 and evaluate its potential as a probiotic in aquaculture by identifying genes related to probiotic functionalities, stress resistance, and interactions with the host and other microorganisms. We performed genome sequencing, gene prediction, functional annotation, comparative genomics, and phylogenetic analysis to achieve these objectives. This study provides valuable genomic information for understanding the probiotic potential of *S. kunmingensis* TFRC-KFRI-1 and contributes to the development of novel probiotic strains for various applications, particularly in aquaculture, where the demand for sustainable and effective alternatives to antibiotics is increasing.

## 2. Materials and Methods

### 2.1. Isolation and Identification of Stutzerimonas kunmingensis TFRC-KFRI-1

*Stutzerimonas kunmingensis* TFRC-KFRI-1 was isolated from the gut of a Manila Clam (*Ruditapes philippinarum*) collected from sediment in the West Sea of South Korea. The clam was collected at a depth of 15 cm using a hand dredge on 23 April 2024. The clam was transported to the laboratory in a cooler with seawater and kept at 4 °C until dissection. The gut of the clam was aseptically dissected using sterile scissors and forceps. The dissected gut was homogenized in 1 mL of sterile seawater using a sterile mortar and pestle. The homogenate was serially diluted to 10^−5^ in sterile seawater. A 100 μL aliquot of the 10^5^ dilution was spread onto Luria–Bertani (LB) agar plates (BD, Franklin Lakes, NJ, USA). The plates were incubated at 30 °C for 48 h. Subsequently, single colonies were isolated from the spread plates by the streaking method on LB agar.

### 2.2. 16S rRNA Gene Sequencing and Analysis

Genomic DNA was extracted from the isolated strain using a commercial DNA e ATL buffer traction kit (Qiagen, Hilden, Germany) according to the manufacturer’s instructions. The 16S rRNA gene was amplified by PCR using the universal primers 27F (5′-AGAGTTTGATCCTGGCTCAG-3′) and 1492R (5′-GGTTACCTTGTTACGACTT-3′). The PCR reaction mixture (50 μL) contained 5 μL of 10× PCR buffer, 1 μL of 10 mM dNTP mixture, 1 μL of each primer (10 pmol/μL), 1.25 U of Taq DNA polymerase (Takara, Kyoto, Japan), 1 μL of template DNA, and sterile distilled water to adjust the final volume. The PCR amplification was performed in a thermal cycler (Bio-Rad, Hercules, CA, USA) with the following conditions: initial denaturation at 95 °C for 5 min, followed by 35 cycles of denaturation at 95 °C for 30 s, annealing at 55 °C for 30 s, and extension at 72 °C for 1 min, and a final extension at 72 °C for 10 min. The 16S rRNA PCR product was purified and sequenced by a commercial sequencing service (Cjbioscience, Seoul, Republic of Korea). The obtained 16S rRNA gene sequence was compared with the EzBioCloud database (https://www.ezbiocloud.net/, Cjbioscience, Republic of Korea) to identify the strain.

### 2.3. Genomic DNA Extraction

Genomic DNA from *Stutzerimonas kunmingensis* TFRC-KFRI-1 was extracted using the Qiagen MagAttract HMW DNA Kit (Qiagen, Germany) following the manufacturer’s protocol. The strain was cultured on Luria–Bertani (LB) agar at 30 °C for 24 h, and a single colony was inoculated into 5 mL of LB broth and incubated at 30 °C for 24 h with shaking at 200 rpm. Cells were harvested by centrifugation (10,000× *g*, 1 min), and the pellet was resuspended in 500 μL of Buffer ATL and 20 μL of Proteinase K. After vortexing, the suspension was incubated at 56 °C for 1 h with vortexing every 15 min. Following RNase A treatment, the lysate was processed with Buffer AL and ethanol, and DNA was purified using magnetic beads. DNA quality was assessed by NanoDrop with a 260/280 ratio between 1.8 and 2.0 and a 260/230 ratio above 2.0, indicating high purity.

### 2.4. Genome Sequencing

The DNA sample used for the Illumina MiSeq TruSeq service was specified as having a concentration of ≥10 ng/µL, a total amount of 400 ng, and an A260/A280 ratio of ≥1.8. The genome of *S. kunmingensis* TFRC-KFRI-1 was sequenced on the Illumina MiSeq platform (Illumina, San Diego, CA, USA), generating 2 × 300 bp paired-end reads using the 600 cycle MiSeq Reagent Kit v3 (Illumina, USA, Cat. No. MS-102-3003) [14]. The sequencing library was prepared using the TruSeq DNA Nano Library Prep Kit (Illumina, USA, Cat. No. 20015964) following the manufacturer’s protocol. Genomic DNA was fragmented to an average size of 550 bp using a Covaris M220 Focused-ultrasonicator™ (Covaris Ltd., Brighton, UK). The Covaris M220 instrument settings, including duty factor, peak incident power, cycles per burst, and treatment time, were optimized to achieve the desired DNA fragment size distribution. Fragmented DNA was quantified using a Bioanalyzer 2100 (Agilent, Palo Alto, CA, USA) with the DNA 7500 kit to ensure an appropriate size distribution and concentration for library preparation.

### 2.5. Library Preparation and Sequencing

The DNA fragments were end-repaired, A-tailed, and ligated to adapters with unique indices using the TruSeq DNA Nano Library Prep Kit. The adapter-ligated DNA was amplified by PCR using the PCR Master Mix and PCR Primer Cocktail from the kit. The PCR conditions were as follows: initial denaturation at 98 °C for 30 s, followed by 8 cycles of denaturation at 98 °C for 10 s, annealing at 60 °C for 30 s, and extension at 72 °C for 30 s, and a final extension at 72 °C for 5 min. The amplified library was purified using AMPure XP beads (Beckman Coulter, Brea, CA, USA, Cat. No. A63881) with a 1.0× bead volume ratio. The quality and concentration of the purified library were assessed using an Agilent 2100 Bioanalyzer (Agilent Technologies, Santa Clara, CA, USA) with the High Sensitivity DNA Kit (Agilent Technologies, USA, Cat. No. 5067-4626). The library size distribution and concentration were confirmed to meet the Illumina sequencing requirements. The prepared library was sequenced on the Illumina MiSeq platform with 2 × 300 bp paired-end reads using the 600 cycle (MiSeq Reagent Kit v3) sequencing kit (Illumina, USA, Cat. No. MS-102-3003).

### 2.6. Data Quality Control and Genome Assembly

The raw sequencing reads were processed for quality control using Trimmomatic v0.36 with the following parameters: ILLUMINACLIP: TruSeq3-PE.fa:2:30:10, LEADING:3, TRAILING:3, SLIDINGWINDOW:4:15, and MINLEN:36, to remove adapter sequences, low-quality bases, and short reads. PhiX control sequences were filtered out using BBMap v38.32 with default settings. The resulting high-quality reads were assembled de novo using SPAdes v3.15.3 [15], optimized for paired-end Illumina data. The quality of the genome assembly was subsequently evaluated with QUAST v5.0.2 [16].

### 2.7. Gene Prediction and Annotation

The identification of protein-coding genes and the functional annotation of the draft genome assembly of *Stutzerimonas kunmingensis* TFRC-KFRI-1 were performed using an integrated set of bioinformatics tools and pipelines. Initially, the assembled contigs were analyzed using the EzBioCloud genome database pipeline, which offers rapid gene prediction and annotation based on a curated bacterial genome database. To further enhance the annotation, the NCBI Prokaryotic Genome Annotation Pipeline (PGAP) v6.8 was employed, leveraging a comprehensive set of algorithms and databases for accurate gene prediction and functional characterization [17].

Gene prediction was performed using Prodigal v2.6.2, which identified protein-coding sequences (CDSs) within the assembled genome by applying advanced algorithms based on intrinsic sequence characteristics, such as codon usage patterns and open reading frame (ORF) lengths [18]. The functional annotation of the predicted CDSs was carried out by comparing them against a broad database of protein families, including the Best-placed Reference Protein set, allowing the assignment of putative functional roles. Additionally, CDSs were categorized into functional groups based on orthologous groups from the EggNOG v5.0 database, providing a detailed overview of the genome’s functional repertoire [19]. To assess the metabolic potential and pathways encoded in the genome, CDSs were mapped to the KEGG database using UBLAST, which revealed various metabolic pathways and functional modules in *S. kunmingensis* TFRC-KFRI-1 [20].

The quality of the genome annotation was evaluated using CheckM v1.2.3, which assesses genome completeness and contamination by comparing the assembly against a set of conserved marker genes specific to the genus *Stutzerimonas* [21]. This integrative approach to gene prediction and annotation thus offers a comprehensive understanding of the coding potential and functional capabilities of *S. kunmingensis* TFRC-KFRI-1.

### 2.8. Phylogenetic Analysis

To determine the phylogeny of *S. kunmingensis* TFRC-KFRI-1 to other *Stutzerimonas* species, a phylogenetic tree was constructed based on 16S rRNA gene sequences. A dataset of 29 sequences, including TFRC-KFRI-1 and related *Stutzerimonas* strains, was obtained from the EzBioCloud database [22]. The 16S rRNA gene sequences were aligned using the MUSCLE algorithm with default parameters [23]. The alignment was then trimmed to remove poorly aligned regions using TrimAl v1.4.rev15 with the “automated1” option [24].

The evolutionary history was inferred using the Maximum Likelihood method implemented in MEGA X [25]. The Tamura–Nei model was selected as the best-fitting nucleotide substitution model based on the Bayesian Information Criterion (BIC) [26]. This model accounts for variations in nucleotide substitution rates among sites and unequal base frequencies. The robustness of the inferred tree was assessed by bootstrapping with 1000 replicates. The final dataset for phylogenetic analysis comprised 1282 nucleotide positions.

The resulting phylogenetic tree provides insights into the evolutionary relationships among *Stutzerimonas* species and places *S. kunmingensis* TFRC-KFRI-1 within the genus. This analysis helps to understand the taxonomic position of the newly isolated strain and its relationship to other known *Stutzerimonas* species.

### 2.9. Pathogenicity Prediction

The pathogenic potential of *Stutzerimonas kunmingensis* TFRC-KFRI-1 was assessed using PathogenFinder v1.1 (https://cge.food.dtu.dk/services/PathogenFinder/), a web-based tool designed to predict bacterial pathogenicity based on genomic data [27]. The assembled genome in FASTA format was submitted to the PathogenFinder platform, which compares protein-coding sequences against a curated database of pathogenic and non-pathogenic bacteria. A machine learning algorithm was employed to estimate the likelihood of human pathogenicity by analyzing specific protein families associated with pathogenicity. The results provide a preliminary evaluation of the strain’s pathogenic potential, guiding future studies on its virulence.

## 3. Results

### 3.1. Genome Sequencing and Assembly

A total of 14,700,428 raw reads were generated from the Illumina MiSeq platform. After quality filtering, 13,069,158 high-quality reads were retained for subsequent analyses. The *S. kunmingensis* TFRC-KFRI-1 genome was assembled into 12 contigs with a total size of 4,756,396 bp and a GC content of 62.8%. The sequencing depth was 231.22× and the N50 value was 1,031,488 (Table 1).

### 3.2. Genome Annotation and Comparative Analysis

The assembled genome was annotated using the NCBI Prokaryotic Genome Annotation Pipeline (PGAP) and the EzBioCloud genome database pipeline [17,28]. A total of 4519 genes were predicted, including 4451 protein-coding sequences (CDSs) and 26 CDSs without predicted protein products. The genome also contained 8 rRNAs (5S: 1, 16S: 4, 23S: 3), 56 tRNAs, and 4 ncRNAs. Comparative genomic analysis revealed that *S. kunmingensis* TFRC-KFRI-1 exhibited an average nucleotide identity (ANI) of 96.87% and a 16S rRNA sequence similarity of 99.45% *S. kunmingensis* HL22-2^T^ (GenBank accession number: GCA_900114065.1).

### 3.3. Genome Map and COG Analysis

**Figure 1:**
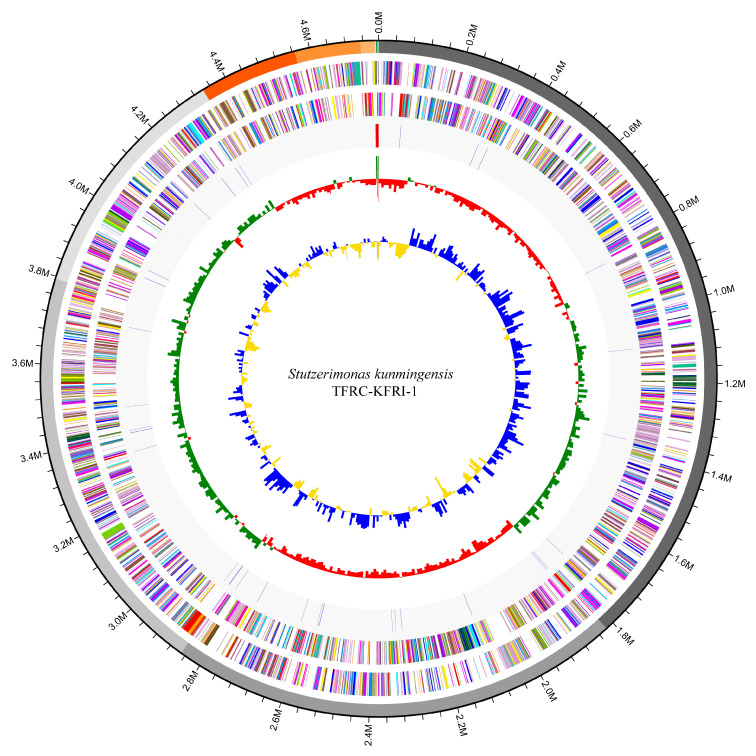
Circular genome map of *S. kunmingensis* TFRC-KFRI-1. The outermost ring represents the genome size in Mb. The second ring shows the 12 contigs, color-coded and arranged by size. The inner rings depict CDSs colored according to COG functional categories. The innermost rings display GC content (green for high and purple for low) and GC skew (yellow for positive and blue for negative).

**Figure 2:**
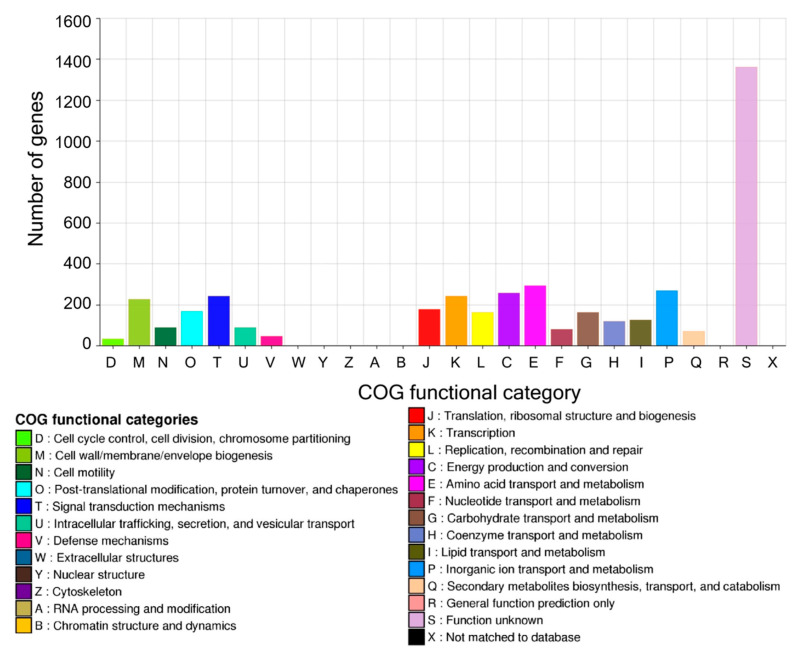
Distribution of COG (clusters of orthologous groups) functional categories in the genome of *S. kunmingensis* TFRC-KFRI-1.

A circular genome map was generated using the CGView Server to visualize the genomic features of *S. kunmingensis* TFRC-KFRI-1 (Figure 1) [29]. The map displays the 12 contigs, ordered by size, with CDSs color-coded according to their functional categories based on the clusters of orthologous groups (COG) classification. The GC content and GC skew are also represented on the map, providing insights into the genomic architecture. COG analysis revealed that the most abundant categories were those associated with replication, recombination, and repair (12.7%); translation, ribosomal structure, and biogenesis (7.8%); transcription (7.6%); carbohydrate transport and metabolism (7.5%); and cell wall/membrane/envelope biogenesis (5.7%), excluding those with unknown function (29.7%) (Figure 2, Table S1).

### 3.4. Phylogenetic Analysis

**Figure 3:**
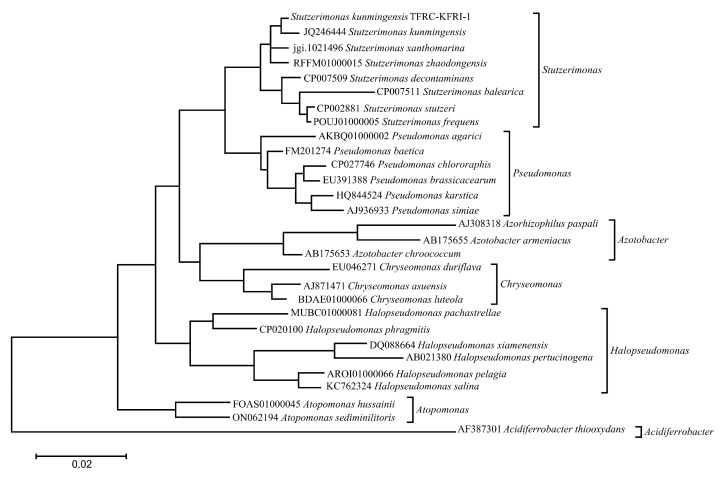
Phylogenetic tree based on 16S rRNA gene sequences showing the relationship of *S. kunmingensis* TFRC-KFRI-1 to other *Stutzerimonas* species. The tree was constructed using the Maximum Likelihood method with the Tamura–Nei model. Bootstrap values (1000 replicates) are shown next to the branches.

Phylogenetic analysis based on 16S rRNA gene sequences placed *S. kunmingensis* TFRC-KFRI-1 in close relation to *S. kunmingensis* JQ246444, suggesting a recent common ancestor and potentially shared phenotypic traits (Figure 3).

### 3.5. Genomic Features

Several genes encoding proteins involved in carbohydrate metabolism were identified in the genome of *S. kunmingensis* TFRC-KFRI-1, including alpha-glucosidase (ACBG90_00780), pullulanase-type alpha-1,6-glucosidase (ACBG90_12020), beta-glucosidase BglX (ACBG90_10810), and beta-glucosidase (ACBG90_04060). Moreover, six genes implicated in glycogen degradation were identified (ACBG90_06815, ACBG90_19440, ACBG90_20935, ACBG90_12020, ACBG90_04215, and ACBG90_07540), highlighting the strain’s metabolic versatility.

Genes associated with lactic acid metabolism were also annotated, including L-lactate permease (ACBG90_00420 and ACBG90_07385) and L-lactate oxidation iron-sulfur protein (ACBG90_00430), which are involved in lactate transport and oxidation, respectively. The genome also harbors genes encoding components of the pyrroloquinoline quinone (PQQ) biosynthesis pathway, including pqqC (ACBG90_20540), pqqD (ACBG90_20545), and pqqE (ACBG90_20550), which are implicated in promoting bacterial growth and antioxidant activity.

Additionally, genes involved in arginine biosynthesis were identified, including argS (ACBG90_07960), argC (ACBG90_08560), argE (ACBG90_06555), and argA (ACBG90_06560). These genes play a critical role in protein biosynthesis and cellular metabolism. Genes associated with prokaryotic defense systems were also discovered, including components of the Type I restriction–modification system, such as restriction endonuclease subunit S (ACBG90_18135), restriction endonuclease subunit R (ACBG90_18170, ACBG90_13245), and modification subunit M (ACBG90_13255). Furthermore, genes related to the Zorya anti-phage system, including zorC (ACBG90_18160) and zorD (ACBG90_18165), were identified, suggesting that the strain harbors sophisticated phage defense mechanisms.

### 3.6. Pathogenicity Prediction

The analysis of the TFRC-KFRI-1 genome using PathogenFinder did not detect any protein families associated with human pathogenicity, suggesting that this strain lacks the genomic features typically found in pathogenic organisms. These findings indicate that *S. kunmingensis* TFRC-KFRI-1 is likely non-pathogenic, supporting its potential application in probiotic formulations and industrial biotechnology.

## 4. Discussion

The genomic analysis of *S. kunmingensis* TFRC-KFRI-1 provides valuable insights into its potential applications in industrial and probiotic settings. This newly isolated strain exhibits a range of metabolic capabilities and defense mechanisms that highlight its adaptability and potential for biotechnological exploitation.

The relatively high GC content (62.8%) of the TFRC-KFRI-1 genome suggests an inherent ability to withstand environmental stress. As observed in previous studies, higher GC content is often correlated with increased resistance to environmental challenges such as high temperature, salinity, and UV radiation [30]. This genomic feature may contribute to the strain’s ability to thrive in diverse environments, including those relevant to industrial processes and aquaculture.

Phylogenetic analysis based on 16S rRNA gene sequences places TFRC-KFRI-1 in close proximity to *S. kunmingensis* JQ246444. This finding, coupled with the observed ANI value of 96.87%, suggests a recent common ancestor and potential similarities in phenotypic traits, including environmental resilience and metabolic versatility [13]. Further investigation into the phenotypic characteristics of TFRC-KFRI-1, particularly in comparison to JQ246444, could reveal valuable traits for biotechnological applications.

The presence of genes encoding carbohydrate-active enzymes (CAZymes) in the TFRC-KFRI-1 genome highlights its potential for bioconversion processes. Specifically, the identified alpha-glucosidase, pullulanase-type alpha-1,6-glucosidase, and beta-glucosidase genes suggest an ability to hydrolyze a variety of polysaccharides, including starch, pullulan, and cellulose. This capability could be harnessed for biofuel production, bioremediation, and other industrial applications that require the breakdown of complex carbohydrates [31].

The identification of genes involved in lactic acid metabolism, namely L-lactate permease and L-lactate oxidation iron-sulfur protein, points to the strain’s potential as a probiotic. Efficient lactate metabolism is a desirable trait in probiotics, particularly those intended for use in food biotechnology and aquaculture, as it can contribute to improved gut health and nutrient absorption [32,33]. Furthermore, the presence of genes involved in PQQ biosynthesis strengthens the probiotic potential of TFRC-KFRI-1. PQQ is known to promote bacterial growth and provide antioxidative defense, contributing to overall health benefits [34].

The ability to synthesize arginine, as evidenced by the presence of arginine biosynthesis genes in the TFRC-KFRI-1 genome, could be valuable for industrial applications. Arginine is a crucial amino acid involved in various metabolic processes, including protein synthesis and nitrogen metabolism [35]. The strain’s capacity for arginine biosynthesis may enhance its applicability in industrial settings where efficient protein production and cellular growth are desired.

The presence of prokaryotic defense systems, including the Type I restriction–modification system and the Zorya anti-phage system, suggests that TFRC-KFRI-1 possesses robust mechanisms to protect against phage attacks [36]. These defense systems are crucial for maintaining genomic integrity and stability in environments where bacteriophages are prevalent. This genomic resilience further supports the potential of TFRC-KFRI-1 for industrial applications, particularly those involving large-scale fermentation or challenging environmental conditions.

In conclusion, genomic analysis has identified *Stutzerimonas kunmingensis* TFRC-KFRI-1 as a resilient microorganism with potential for industrial and probiotic applications. This strain possesses genes for carbohydrate metabolism, lactic acid utilization, arginine synthesis, and phage defense. Experimental validation is needed to confirm its potential in biofuel production, bioremediation, aquaculture, and probiotic development. Future research, including functional studies and comparative analyses with related strains, will be essential to fully explore the biotechnological potential of *S. kunmingensis* TFRC-KFRI-1.

## Figures and Tables

**Table 1:** Genome features of *S. kunmingensis* TFRC-KFRI-1 and sequencing assembly statistics.

Feature	Value
Raw reads	14,700,428
Filtered reads	13,069,158
Genome Length (bp)	4,756,396
GC content (%)	62.8
Sequencing depth (×)	231.22
Number of Contigs	12
N50 (bp)	1,031,488
tRNA	56
rRNA (5S, 16S, 23S)	1, 4, 3 (0, 3, 2) *
ncRNA	4
Total genes	4519
CDS	4425
Completeness (%)	99.67 **
Accession Number	JBGJJB000000000.1.

* Number of partial genes. ** CheckM analysis.

## Data Availability

The data presented in this study are available in the NCBI database. The genome sequence of *Stutzerimonas kunmingensis* TFRC-KFRI-1 has been deposited under GenBank accession number [JBGJJB000000000.1]. Additional project information can be found under BioProject [PRJNA1147901] and Bio-Sample [SAMN43173893].

## Supplementary Materials

The following supporting information can be downloaded at: https://www.mdpi.com/article/10.3390/microorganisms12122402/s1, Table S1: *Stutzerimonas_kunmingensis*_TFRC-KFRI-1_CDSs.
